# Simultaneous Quantitative Determination of Metoprolol, Atorvastatin and Ramipril in Capsules by a Validated Stability-Indicating RP-UPLC Method

**DOI:** 10.3797/scipharm.1004-14

**Published:** 2010-08-24

**Authors:** Raja Kumar Seshadri, Makarand Madhukar Desai, Thummala Veera Raghavaraju, Deepa Krishnan, Dama Venugopala Rao, Ivon Elisha Chakravarthy

**Affiliations:** 1 Analytical Research and Development, Integrated Product Development, Dr. Reddy’s Laboratories Ltd., Bachupally, Hyderabad-500 072, India; 2 Department of Chemistry, Rayalaseema University, Kurnool-518002, A.P., India

**Keywords:** Fixed-dose combination, Chromatography, Method Validation, Reviro, Impurities

## Abstract

A simple ultra performance liquid chromatographic (UPLC) method has been developed for the simultaneous estimation of Metoprolol (MT), Atorvastatin (AT) and Ramipril (RM) from capsule dosage form. The method was developed using Zorbax® XDB-C18 (4.6 mm × 50 mm, 1.8 μm) column with a mobile phase consisting of 0.06% ortho phosphoric acid in Milli Q® water having an ion pair reagent, 0.0045 M Sodium lauryl sulphate as buffer, at ratio of buffer: Acetonitrile (50:50 v/v), at 55°C column temperature with a flow rate of 1.0 ml/min. Detection was carried out with ultra-violet detection at 210 nm for RM, MT and AT respectively. The retention times were about 1.3, 2.1 and 2.6 min for MT, AT and RM respectively, the method was validated for linearity, accuracy, precision, specificity, robustness and ruggedness. The % mean recoveries are 101.9, 102.1 and 101.4 for MT, AT and RM respectively. The method was found to be rugged and robust and can be successfully used to determine the three drugs and its combinations.

## Introduction

**Fig.1.**
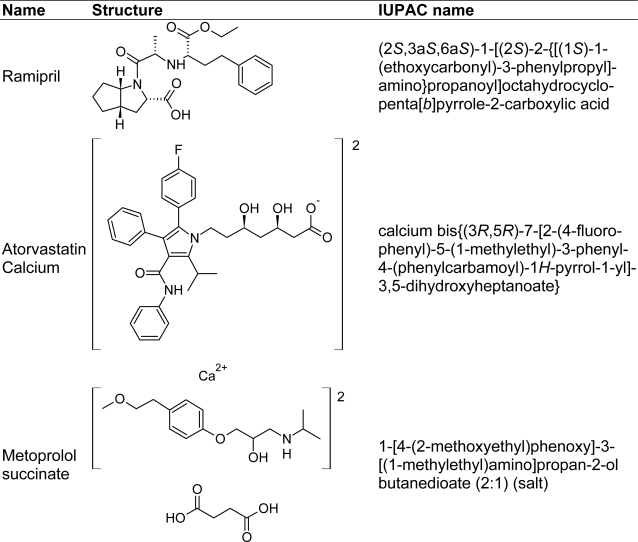
Chemical structures of RM, AT and MT

Ramipril (RM) is a potent and specific angiotensin-converting enzyme (ACE) inhibitor that catalyzes the conversion of angiotensin I to the vasoconstrictor substance, angiotensin II which also simulates the secretion of aldosterone by adrenal cortex leading to vasopressor activity. Thus role of these ACE inhibitors is to inhibit the last step of the biosynthesis of angiotensin II and therefore causing a general vasodilatation and lowering of blood pressure [1–3]. Atorvastatin calcium (AT) [4] is a lipid lowering drug [5, 6], used for treatment of hypercholesterolemia. Metoprolol succinate (MT) acts as a beta-adrenergic blocking agent, which reduces chest pain and lowers high blood pressure [2, 7].The chemical structures of RM, AT and MT are shown in Fig. 1.

Many dosage forms of Metoprolol, Atorvastatin and Ramipril are available in market as a single or as combination dosage form with other drugs for effective therapy. As an individual molecule Atorvastatin shows effectiveness in lowering lipid levels in condition of dyslipidemia and metabolic syndrome. But since the hypercholesterolemia is also recognized as a causative factor in the development of atherosclerosis and hypertension, treatment with a beta-adrenergic blocking agent Metoprolol which is known to reduce the chest pain and lowering the high blood pressure also have antiatherosclerotic effects and Ramipril ACE (angiotensin converting enzyme) will always be a beneficial point for the patients by controlling hypertension, angina associated with dyslipidemia and metabolic syndrome. Hence these three combination formula is developed as once daily dosage form.

Dr. Reddy’s is the first pharmaceutical company to develop this unique three-in-one combination product containing RM, AT and MT which is unique and has got the approval from Drug Controller General of India (DCGI), the Brand name of the product is Reviro.

Literature survey revealed several analytical methods such as UV Spectrophotometer [8–10], Liquid Chromatography [11–24], HP-TLC [25], LC-MS [26], UPLC [27, 28] Spectrofluorimetry [29, 30] have been reported for the determination of Metoprolol, Atorvastatin and Ramipril individually and in combination with different pharmaceutical dosage forms and biological samples [31]

Till date, to the best of our knowledge, no method has been reported in the literature for simultaneous determination of MT, AT and RM in oral dosage formulation. It is felt necessary to develop a stability indicating assay method for three compounds simultaneously.

Metoprolol impurity C is official in British Pharmacopoeia [32], Metoprolol diol impurity is a degradation product identified and completely characterized by using IR, Mass and NMR. Ramipril impurities D, E and K are official in British Pharmacopoeia [33]. Atorvastatin impurities lactone and hydroxyl epoxy oxazinine are the degradation products, isolated and characterized inhouse by using IR, Mass and NMR.

## Results and Discussion

### Optimization of chromatographic conditions

The HPLC method is optimized with a view to develop a stability indicating Assay. A stability-indicating assay method should accurately measure the active ingredients, without interference from degradation products, process impurities, excipients, or other potential impurities. The key objective of the method is to get the separation of all potential impurities of MT, AT and RM from their analyte peaks. Pure drug along with its related impurities were injected in Ramipril capsules BP method, it was observed that Atorvastatin peak shape is not symmetric and Atorvastatin major degradants Lactone and Hydroxyl epoxy oxazinine eluted after 20 min. Subsequently Metoprolol tablets USP method was evaluated for separation of RM, AT and MT and its respective impurities. In the metoprolol method, the Atorvastatin peak eluted at 91 min and also peak fronting is observed for Ramipril, Atorvastatin peaks.

To develop a new method, the pure drug along with its related impurities were injected in different solvent systems containing sodium lauryl sulfate and orthophosphoric acid along with various ratios of organic modifiers acetonitrile and methanol on a C8 column. The impurities and degradants pertaining to RM, AT and MT active moieties were monitored at wavelength of 210 nm. Further, 0.06% ortho phosphoric acid in Milli-Q water was used as buffer having an ion pair reagent, 0.045 M sodium lauryl sulfate in the mobile phase to achieve effective separation between impurities and the analyte peaks. Buffer and acetonitrile in the ratio 50:50 at 1.0 ml/min flow at ambient temperature; it was observed that impurity E and impurity D peaks of Ramipril were merging with Metoprolol and Atorvastatin peaks. Modification of the buffer and acetonitrile ratio to 55:45 resulted the separation of impurity E and impurity D from Metoprolol and Atorvastatin peaks, but Ramipril peak was asymmetric i.e. peak tailing observed more than 2.0. The increase in the column temperature from ambient to 40°C resulted in achieving the peak symmetry and the separation all three analyte peaks AT, RM and MT from impurity peaks, but the observed runtime is more i.e. 45 min.

To reduce the runtime further, the above HPLC method is verified in UPLC using Zorbax® Eclipse plus-C8, 2.1 mm × 50 mm, 1.8 μm with flow rate of 0.6 ml/min. On UPLC, the runtime is reduced to 10 minutes but the peak symmetry was failing for Metoprolol peak i.e. tailing more than 2.0. To improve the peak symmetry for MT, column was changed to Zorbax® XDB-C18, 4.6 mm × 50 mm, 1.8 μm with a flow rate 1.0 ml/min, and column temperature is maintained at 55°C. The chromatographic separation was achieved by following isocratic program using buffer as 0.06% ortho phosphoric acid in Milli Q® water having an ion pair reagent 0.045 M sodium lauryl sulfate; buffer and acetonitrile in the ratio of 50:50 (v/v). The retention times of MT, AT and RM are 1.324 min, 2.148 min and 2.684 min respectively. This method is capable to separate all impurities (two known degradants of Atorvastatin, four known degradants of Ramipril and two known degradants of Metoprolol) from its analyte peaks within 5 minutes.

**Fig. 3.**
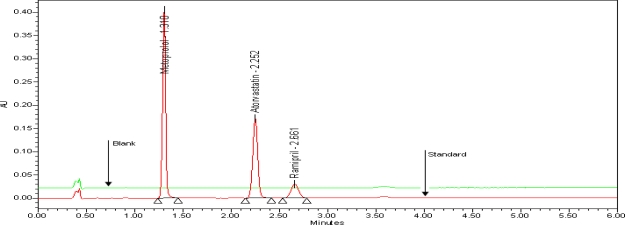
Overlay chromatogram of blank and standard, extracted at 210nm for RM, AT and MT

**Fig. 4.**
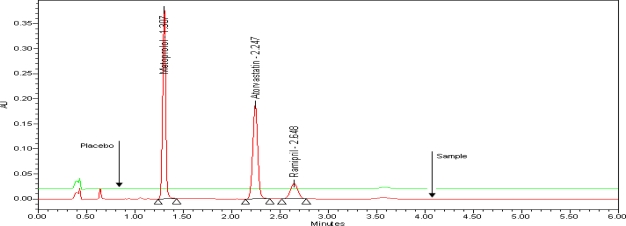
Overlay chromatogram of Placebo and Test, extracted at 210nm for RM, AT and MT

**Fig. 5.**
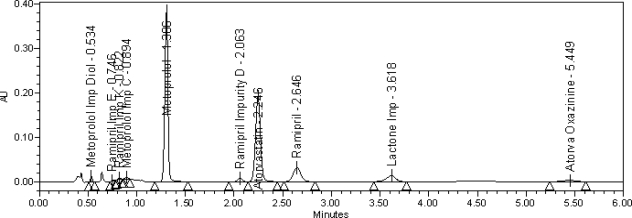
Spiked chromatogram of AT, RM and MT along with impurities.

The typical overlay chromatogram of blank and standard is shown in Fig. 3. The typical overlay chromatogram of placebo and test is shown in Fig. 4. The Spiked chromatogram of AT, RM and MT along with impurities is shown in Fig. 5.

The system suitability parameters (see Table 1) indicate that the method meets the preliminary requirements and hence ready to be subjected for validation.

The details of method validation, as per ICH guidelines [34] are presented in the following sections.

### Method Validation

#### Specificity

The samples have been subjected to degradation such as refluxed with 0.1N HCl for 30 min at 60°C, refluxed with 0.1 N NaOH for 30 min at 60°C, refluxed with 3% peroxide for 30 min at 60ºC, exposed to dry heat at 105°C for 15 h, refluxed for 1 hour at 60°C in water, exposed to visible light of 1.2 million lux hours, UV light of 200 watt hours m^−2^ and exposed to 90% relative humidity at 25°C for 7 days (Table 2).

**Fig. 6.**
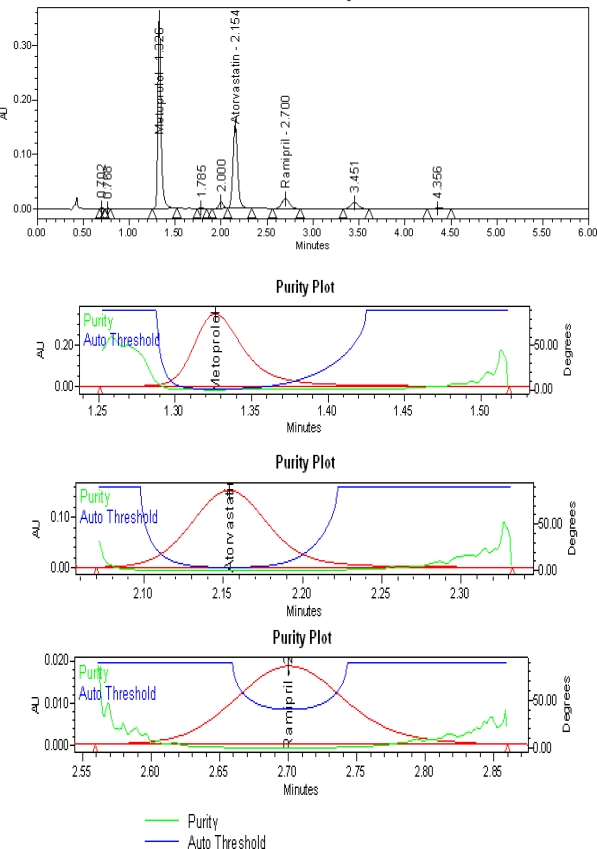
Chromatogram and purity plots of heat stressed MT, AT and RM capsules test

Degradation was not observed in visible light, UV, humidity and water hydrolysis stress studies. Significant degradation was not shown in acid hydrolysis, base hydrolysis and oxidative conditions. However, thermal stress showed significant degradation. It is interesting to note that all the peaks due to degradation are well resolved from the peaks of MT, AT &RM (Fig. 6). Further the peak purity of MT, AT & RM was found to be homogeneous based on the evaluation parameters such as purity angle and purity threshold using Waters Empower Networking Software. The verification of peak purity indicates that there is no interference from degradants, facilitating error-free quantification of MT, AT and RM. Thus, the method is considered to be “Stability-indicating”.

#### Linearity

The linear regression analysis of MT, AT and RM was constructed by plotting the concentration of each analyte versus peak area. The calibration curves were linear in the range of 105–840 μg/ml, 20–160 μg/ml and 10–80 μg/ml for MT, AT and RM respectively. The linear regression equations are given below.

$$\begin{array}{l} \text{MT}:\;\text{Y}=2033.264\;\text{conc}.+5139.971\;({\text{r}}^{2}=0.9996)\\ \text{AT}:\;\text{Y}=7928.515\;\text{conc}.+5806.824\;({\text{r}}^{2}=0.9998)\\ \text{RM}:\;\text{Y}=4045.254\;\text{conc}.+339.373\;({\text{r}}^{2}=0.9995) \end{array}$$

#### Precision

The precision of test method was evaluated by analyzing six samples of MT, AT and RM capsules. The % RSD of assay of three analytes during precision was found to be less than 2%. The results are shown in Table 3, which indicates that the method is precise.

#### Accuracy

Recovery study of MT, AT and RM from spiked placebo is conducted. Samples are prepared by mixing placebo with MT, AT and RM raw material equivalent to about 20%, 50%, 80%, 100%, and 160% of the assay of nominal sample concentration. Sample solutions are prepared in triplicate for each spike level as described in the sample preparation. The % mean recoveries of individual analyte from formulation samples were found to be satisfactory. The summary of % recovery was mentioned in Table 4.

#### Robustness

To determine the robustness of the developed method, experimental conditions are deliberately altered one factor after the other. The effect of change in flow rate (± 0.2 ml), organic phase composition (± 5%) and column temperature (± 5°C) on the retention time, resolution and asymmetric factor were studied. In all the studies the resolution between MT and AT, AT and RM peaks is greater than 2.5, asymmetric factor is less than 2.0 and theoretical plates are more than 2500 for MT, AT and RM peaks, which illustrates the robustness of the method.

#### Test solution stability

The bench top solution stability of test preparation and standard preparation of MT, AT and RM was carried out up to 48 hours, no significant change is observed in % assay of MT, AT and RM. Hence the test solutions are stable upto 48 hrs.

## Experimental

### Reagents and Materials

Active pharmaceutical ingredient of MT, AT and RM and it’s impurities were procured from bulk manufacturers of Dr Reddy’s Laboratories. Capsule dosage form developed by Dr Reddy’s Laboratories, India. HPLC grade acetonitrile was purchased from Merck, Germany. Analytical reagent sodium lauryl sulfate and ortho phosphoric acid were purchased from Merck, Germany. High pure water was prepared by using Millipore Milli Q® plus purification system.

### Apparatus and chromatographic conditions

The Waters Acquity UP-LC system with a photo diode array detector was used for method development, validation and forced degradation studies. The output signal was monitored and processed using Empower software.

### Chromatographic conditions

The chromatographic column used was a Zorbax® XDB-C18, 4.6 mm * 50 mm, 1.8 μm particles. The buffer used was 0.0045 M of sodium lauryl sulfate with 0.06% (1 gm) ortho phosphoric acid. Buffer and acetonitrile in the ratio of 50:50 was used as mobile phase. The flow rate of the mobile phase was 1.0 ml/min. The column was maintained at 55 °C and the wavelength of 210 nm for detection of MT, RM and AT. The injection volume was 2 μL.

### Preparation of standard solution

Standard solution was prepared in methanol containing 420 μg ml^−1^ of Metoprolol succinate, 80 μg ml^−1^ of Atorvastatin and 40 μg ml^−1^ of Ramipril. Purity of standards used is 99.3% as Metoprolol Succinate, 99.3% as Atorvastatin and 99.8% as Ramipril.

### Preparation of Test solution

Contents of 10 capsules along with capsule shell (each capsule containing 47.5 mg MT, 10mg AT and 5 mg RM) was transferred into 250 ml dried volumetric flask, 150 ml of methanol is added and sonicated for 30 min with intermediate shaking (maintain the sonicator temperature between 20°C–25°C), followed by shaking of 15 min. The flask is to allowed cool down to room temperature and then diluted to volume with methanol. A part of solution is centrifuged to get clear solution. Further 5 ml of clear centrifuged solution is transferred into 25 ml volumetric flask and diluted to volume with methanol to obtain sample solution concentration of 420 μg/ml, 80 μg/ml and 40 μg/ml respectively.

## Conclusions

The developed chromatographic assay fulfilled all the requirements to be identified as reliable and feasible method, including accuracy, linearity, recovery and precision data. It is a highly specific and precise analytical procedure and its chromatographic run time of six minutes allows the analysis of a large number of samples in a short period of time. Therefore, this HPLC method can be used as a routine sample analysis as well as stability testing. The method validation shows satisfactory data for all the method validation parameters tested. The developed method is stability-indicating and can be used for quantifying Metoprolol, Atorvastatin and Ramipril in capsule dosage form and their combinations (i.e. MT+AT; MT+RM, AT+RM and AT+RM+MT).

## Figures and Tables

**Fig. 2.**
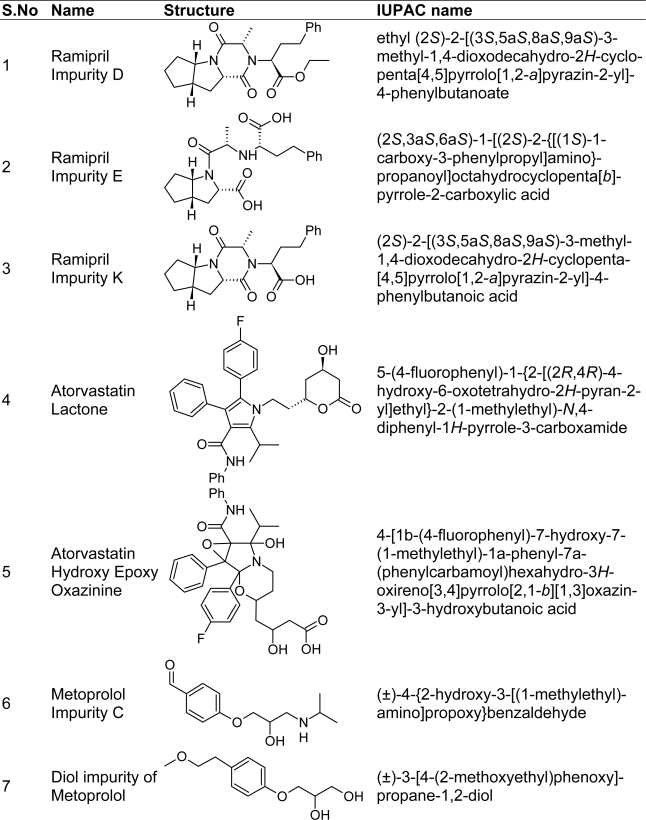
Chemical structures of impurities of RM, AT and MT

**Tab. 1.** System suitability results

**System suitability parameters**	**Metoprolol**	**Atorvastatin**	**Ramipril**
Retention times min	1.310	2.252	2.661
Theoretical plates	7638	8581	4958
Asymmetric factor	1.5	1.1	1.1
Resolution	–	10	4

**Tab. 2.** Forced degradation data

**Degradation conditions**	**% Degradation**		
	**Metoprolol**	**Atorvastatin**	**Ramipril**
Exposed to Visible light for about 1,200 K lux	0.2	0.9	1.5
Exposed to UV for 200 Watt hours m^−2^	0.1	0.6	0.3
Exposed to humidity at 25 °C, 90% RH for about 7 days	0.0	1.2	1.4
Refluxed with purified water for about 1 hour at 60 °C	0.1	0.9	0.7
Refluxed with 0.1 N HCI solution for about 30 min at 60 °C	0.0	1.2	2.7
Refluxed with 0.1 N NaOH solution for about 30 min at 60 °C	0.1	0.7	2.6
Refluxed with 3% H_2_O_2_ solution for about 30 min at 60 °C	0.3	2.2	4.8
Exposed to dry heat for about 15 hours at 105 °C	0.4	32.3	33.5

**Tab. 3.** Precision of the method

**S.NO.**	**% Assay**		
	**Metoprolol**	**Atorvastatin**	**Ramipril**
1.	101.9	102.4	102.2
2.	101.5	101.7	100.4
3.	102.0	102.0	101.7
4.	101.3	102.3	102.3
5.	102.3	102.1	100.4
6.	102.3	102.3	101.5
Average	101.9	102.1	101.4
% RSD	0.4	0.3	0.8

**Tab. 4.** Accuracy of the method

**Accuracy level**	**MT Added (mg)**	**MT Recov.^a^ (%)**	**Mean MT (%)**	**AT Added (mg)**	**AT Recov.^a^ (%)**	**Mean AT (%)**	**RM Added (mg)**	**RM Recov.^a^ (%)**	**Mean RM (%)**
20%	99.960	101.0		20.565	101.6		10.283	100.6	
50%	246.954	101.8		52.036	100.2		26.018	102.0	
80%	392.084	102.1	101.8	80.700	102.5	101.8	40.350	101.4	101.2
100%	495.629	102.1		102.731	102.4		51.366	101.0	
160%	783.014	102.0		162.870	102.4		81.435	101.0	

a Mean for three determinations at each level.
